# Granulomatous inflammation in pulmonary pathology of 2019 novel coronavirus pneumonia: case report with a literature review

**DOI:** 10.1186/s42047-020-00071-2

**Published:** 2020-09-16

**Authors:** Elif Usturalı Keskin, Ebru Tastekin, Nuray Can, İnci Usta, Nermin Tuncbilek, Derya Karabulut, Osman Kula, Onur Kırkızlar

**Affiliations:** 1 Trakya University Faculty of Medicine, Department of Pathology, Edirne, Turkey; 2 Trakya University Faculty of Medicine, Department of Radiology, Edirne, Turkey; 3 Trakya University Faculty of Medicine, Department of Hematology, Edirne, Turkey

**Keywords:** COVID-19, Lung biopsy, Lung CT images, Lung histopathology images

## Abstract

**Background:**

Coronavirus disease 2019 (COVID-19), which started in Wuhan, China, in late December 2019, was declared a pandemic, infecting more than twelve million people worldwide. Few studies have reported the findings of lung biopsies in COVID-19. Here, granulomatous inflammation was reported for the first time in COVID-19 lung biopsy.

**Case presentation:**

A 54-year-old woman presented to a primary care facility with fever, dry cough, and fatigue. Antibiotherapy was administered for 10 days with the diagnosis of upper respiratory tract infection. However, her condition did not improve and she was admitted to the hospital. In physical examination, crepitant rales were heard in both lungs. Anemia and thrombocytopenia were detected in laboratory tests and she was referred to the hematology clinic. Bone marrow aspiration and flow cytometry showed she had acute myeloid leukemia. Computed tomography-integrated positron emission tomography with a history of previous breast cancer revealed a heterogeneous mass-like lesion in the left lung. The primary malignancy could not be ruled out and tru-cut biopsy was performed. Tests for tuberculosis were negative. Throat swab sample was taken and a real-time polymerase chain reaction confirmed that she had COVID-19. Radiological findings were evaluated as the progression of COVID-19 pneumonia on computed tomography 6 days after biopsy. Alveolar damage, edema, vascular congestion, mild inflammatory infiltration, type-2 pneumocyte hyperplasia, interstitial fibrosis, early fibrotic changes, fibrinous, organized pneumonia pattern, noncaseating granulomatous inflammation, and desquamation in alveolar epithelial cells were noted in lung biopsy.

**Conclusions:**

There were only a few case reports that described lung biopsy findings in COVID-19 at the time of manuscript preparation. This was the first case of noncaseating granulomatous inflammation described in a COVID-19 case.

## Introduction

Coronavirus disease 2019 (COVID-19), which started in Wuhan, China, in late December 2019 and progressed with severe acute respiratory distress syndrome, has since spread to Asia, Europe, and North America, infecting more than twelve million people worldwide (Tian et al. 2020). COVID-19 patients most frequently present with clinical symptoms such as fever, fatigue, dry throat, cough, and more rarely with sputum, headache, diarrhea (Huang et al. 2020; Wang et al. 2020). The number of confirmed cases with severe acute respiratory distress syndrome coronavirus 2 (SARS-CoV-2) infection, the viral agent leading to COVID-19, has reached 12.964,809 worldwide, including 570,288 deaths as of July 14, 2020 (new reference website of world health organization). Deceased patients are often older patients with an underlying disease. Several studies communicated the characteristic clinical and radiological findings in COVID-19 patients (Huang et al. 2020; Wang et al. 2020; Bernheim et al. 2020). Although the number of deaths is quite high, invasive procedures could not be applied due to the highly contagious feature of COVID-19. At the time of manuscript preparation for this report, there were a few studies that included findings of lung biopsy in COVID-19 patients (Tian et al. 2020; Xu et al. 2020; Zhang et al. 2020). Interestingly, we encountered the pathological findings of this new infection in a biopsy taken from the lung mass, although it is not due to COVID-19.

## Case report

**Fig. 1:**
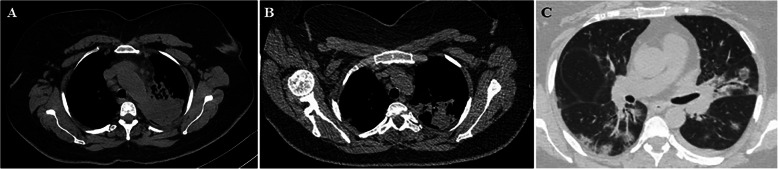
CT imaging findings; **a** A mass like lesion was shown in the left upper lobe apicoposterior segment. **b** Dimensional regression of the mass like lesion and a consolidated area containing air bronchograms was observed in the left upper lobe apicoposterior segment 6 days later. **c** Follow-up CT scan 6 days later showed typical COVID-19 findings with multifocal segmental ground glass opacity infiltration and consolidation with air-bronchogram along the bronchovascular bundles and in subpleural areas with associated interlobular septal thickening and minimal pleural effusion in the left lower lobe

A 54-year-old woman presented to a primary care facility with fever, dry cough, and fatigue. Antibiotherapy was administered for 10 days with the diagnosis of upper respiratory tract infection. However, her condition did not improve and she was admitted to the hospital. In physical examination, crepitant rales were heard in both lungs. Her oropharynx had a natural appearance, and lymphadenopathy was not detected in the neck. Laboratory tests indicated anemia and thrombocytopenia; and she was directed to the hematology clinic. In her peripheral smear, 50–60% myeloid blast cells were detected. A bone marrow biopsy was performed. Bone marrow aspiration and flow cytometry showed approximately 25% blasts and was reported as acute myeloid leukemia transformed from myelodysplastic syndrome. Her medical history revealed that she was diagnosed with left breast cancer 6 years ago, underwent breast-conserving surgery, and had received chemotherapy and hormone therapy. She also had type-2 diabetes. The patient was on clinical follow-up and considered to be in remission. Computed tomography-integrated positron emission tomography (PET/CT) revealed a heterogeneous mass-like lesion with increased FDG (fluorodeoxyglucose) in the left upper lobe apicoposterior segment, with a size of 72 × 63 mm. Although the lung lesion was considered primarily to favor tuberculosis, the primary malignancy of the lung could not be ruled out, and a tru-cut biopsy was performed from the lesion (Fig. 1a). Cell culture and adenosine deaminase test for tuberculosis were negative. After taking the biopsy, throat swab sample was taken, and real-time polymerase chain reaction (RTPCR) analysis confirmed that the patient had COVID-19. Unlike the previous imaging study, computed tomography (CT) that was performed 6 days after biopsy, showed partial regression in the mass-like lesion in the upper lobe of the left lung (Fig. 1b). Besides that, multiple ground glass opacities and irregular nodular consolidations with air-bronchogram along the bronchovascular bundles and linear opacities was revealed especially in the bilateral lung lower lobes (Fig. 1c). Radiological findings were evaluated as progression of COVID-19 pneumonia.

She was immediately admitted to the isolation ward in pandemic service. She was given hydroxychloroquine, antiviral (oseltamivir), azithromycin, and favipiravir. Her vital parameters worsened and she was admitted to the intensive care unit and put on a mechanical ventilator; she died 8 days later.

**Fig. 2:**
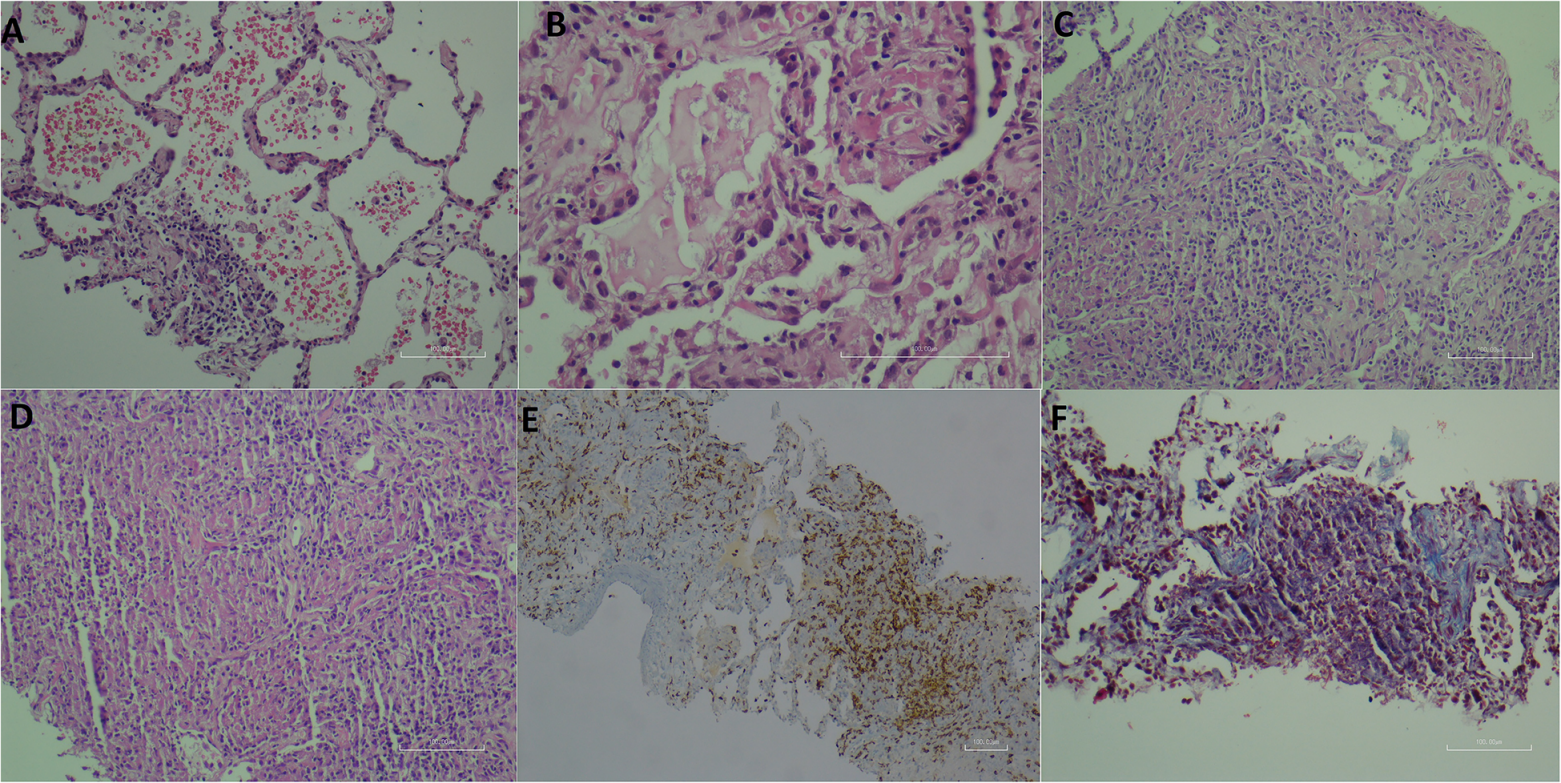
Histopathological changes in the lung in COVID-19 pneumonia; **a** Alveolar damage, congestion, inflammation (HE × 100); **b** pneumocyte hyperplasia, interstitial fibrosis (HE × 400), **c** early fibrotic changes, fibrinous, organized pneumonia (HE × 100), **d** noncaseating suppurative granulomatous inflammation with neutrophils (HE × 100), **e** inflammation consisted rich in CD3 positive lymphocytes (HE × 40), **f** interstitial fibrosis was confirmed with Masson Trichrome histochemical stain (HE × 100)

Malignancy was not considered in hematoxylin-eosin sections prepared from lung biopsy material. Alveolar damage, edema, vascular congestion, and mild inflammatory infiltration were noted (Fig. 2a). Type-2 pneumocyte hyperplasia, interstitial fibrosis, early fibrotic changes, and fibrinous and organizing pneumonia patterns were detected (Fig. 2b, c). Noncaseating granulomatous inflammation with neutrophils and desquamation in alveolar epithelial cells was noted (Fig. 2d). Inflammation consisted of mononuclear cells rich in CD3 positive lymphocytes and neutrophils (Fig. 2e). Pulmonary interstitial fibrosis was confirmed with Masson’s trichrome histochemical stain (Fig. 2f). The periodic acid-Schiff histochemical staining showed no evidence to suggest bacterial or fungal infections. Pathological findings were considered to be consistent with the histopathological findings of COVID-19 pneumonia based on limited studies available in the literature (Tian et al. 2020; Xu et al. 2020; Hwang et al. 2005).

## Discussion and conclusions

Novel coronavirus pneumonia (COVID-19) is the disease caused by the viral agent SARS-CoV-2. SARS-CoV-2 has initially caused an epidemic in Wuhan, China, in late December 2019 and later spread to all continents, infecting more than twelve million people and causing more than 500 thousands deaths in six and half months (new reference website of world health organization). The knowledge about the epidemiological, clinical, and even radiological imaging findings for the disease is growing with new studies. However, due to the risk of high transmission, published autopsy findings are rare, and a few case reports describing the findings of lung biopsy in COVID-19 were available at the time of manuscript preparation for this report (Tian et al. 2020; Xu et al. 2020).

Our patient was immune-suppressed, had a history of breast cancer, and currently had acute myeloid leukemia. She did not have a history of a trip abroad or a contact with a sick person. However, we thought that she might have infected by another patient in the hospital while the underlying cause of bicytopenia detected in the laboratory test results was being investigated. In the patient, alveolar damage, alveolar edema, vascular congestion, and inflammatory cell infiltration were noted in the lung biopsy, similar to those in previous studies. Inflammation was made up of dominant mononuclear cells from lymphocytes and neutrophils. Noncaseating granulomatous inflammation was described for the first time in a COVID-19 case. Interstitial fibrosis and early fibrotic changes were also observed. The findings were consistent with the late-stage pathological findings of COVID-19 pneumonia and were evaluated as diffuse alveolar damage (Hwang et al. 2005). Separately, a case report by Tian et al. described intra-alveolar spherical globules and suspicious viral inclusions (Tian et al. 2020). Xu et al. observed desquamation and hyaline membrane formation in pneumocytes, and the prominent nucleoli cells in the intra-alveolar space were interpreted as other viral cytopathic effects (Xu et al. 2020). In the lung biopsy presented by Zhang et al., shedding of alveolar epithelial cells, type-2 pneumocytic hyperplasia, intra-alveolar fibrinous exudate, interstitial fibrosis, and chronic inflammatory infiltrate were observed similar to our case, and also there was an appearance of organized pneumonia with intra-alveolar fibrous plaques (Zhang et al. 2020). In studies of the SARS-coronavirus outbreak, the findings in SARS-CoV-positive patients were categorized as the early findings in the first 2 weeks of the disease and the late findings after the 14th day. Acute fibrinous exudate showing lung damage was observed more frequently in the early period, while organized exudate and pneumocyte hyperplasia were significantly more common in the late period (Hwang et al. 2005). Based on this categorization, our patient was in the late period.

In thorax CT performed 6 days after the first CT; bilateral irregular opacities, nodular consolidations, and ground-glass opacities were observed, which belonged to late-stage imaging findings of COVID-19 pneumonia progression. Bernheim et al. categorized 121 patients with COVID-19 pneumonia symptoms based on the CT findings as early (2 days after the onset of symptoms), intermediate (3-5 days after), and late group (6-12 days after). Accordingly, the coexistence of bilateral lung involvement, nodular consolidation, and ground-glass opacities mostly accompanies the late imaging findings (Bernheim et al. 2020).

Noncaseating granulomatous inflammation in COVID-19 was described for the first time in this case. Multisystemic granulomatous inflammation in ferrets carrying coronavirus antigen has been described in the literature (Martínez et al. 2008). Also, pyogranulomatous infiltration was observed in the domestic ferret carrying coronavirus (Lindemann et al. 2016). Therefore, granulomatous inflammation in coronavirus infection has been shown in animal studies in the literature and it is not surprising to see it in human cases (Martínez et al. 2008; Lindemann et al. 2016). We considered other infectious agents causing acute/chronic pneumonia and tuberculosis in the differential diagnosis of COVID-19 pneumonia. Separately, an association between COVID-19 and tuberculosis has been described by a cohort study (Tadolini et al. 2020). Before the tests were concluded, we thought that our patient most likely had tuberculosis accompanying COVID-19. However, cell culture and adenosine deaminase test results were negative. Tuberculosis bacilli were not observed in the Ehrlich Ziehl-Neelsen histochemical stain and PCR. Radiological findings were compatible with viral pneumonia rather than tuberculosis. Granulomas were suppurative, not caseified, like tuberculosis granulomas. Of course, we still cannot rule out the presence of tuberculosis bacilli that cannot be detected by these tests. Epidemiological and clinical findings, typical findings in computed tomography, and RTPCR testing supported the diagnosis of COVID-19 pneumonia. The biopsy findings of this newly encountered disease are still unclear. This issue will be better understood as studies report a larger number of cases with lung biopsy findings in COVID-19 patients.

## Data Availability

The data set supporting the conclusions of this article is included within the article. The detail of data analysed during the current case report are not publicly available due to patient privacy but are available from the corresponding author on reasonable request.

## Abbreviations

COVID-19: Coronavirus disease 2019
PET/CT: Computed tomography-integrated positron emission tomography
FDG: Fluorodeoxyglucose
RTPCR: Real time polymerase chain reaction
CT: Computed tomography
SARS: Serious acute respiratory distress syndrome
